# Prevalence and Predictive Factors of Panic Disorder among Adults in Saudi Arabia: A Cross-Sectional Study

**DOI:** 10.1007/s44197-024-00208-6

**Published:** 2024-02-19

**Authors:** Ahmed Aljadani, Khalid Alshammari, Mossa Alshammari, Sulaiman Alshammari, Ahmed Alhuwaydi, Mohamed AbouZed, Islam Shabaan, Nasr Elzahab, Abdullah Altuhayni, Naif Alghasab

**Affiliations:** 1 Internal Medicine Department, Division of Psychiatry, University of Hail, Hail, Saudi Arabia; 2 Internal Medicine Department, Division of Psychiatry, Jouf University, Sakaka, Saudi Arabia; 3 Psychiatry Department, Faculty of Medicine, Al-Azhar University, Cairo, Egypt; 4 Psychiatry Department, Eradah Mental Complex, Hail, Saudi Arabia; 5 Internal Medicine Department, University of Hail, Hail, Saudi Arabia

**Keywords:** Panic Disorder, Panic Attacks, Anxiety Disorders, Prevalence, Risk Factors, Saudi Arabian Adults

## Abstract

Panic disorder (PD) is a severe anxiety disorder characterized by recurrent and unexpected panic attacks that cause intense distress. Despite the high prevalence of panic disorder and its significant impact on life, limited research has been conducted on its prevalence and their associated factors in Saudi Arabia. This study seeks to contribute to the understanding of PD among adults in Saudi Arabia by examining its prevalence and associated factors, using an online survey method. A validated questionnaire-based cross-sectional study was conducted targeting 1276 Saudi adults. Data were collected electronically via Google Forms from the eligible participants. The questionnaire comprised three sections: sociodemographic information, medical history, and a validated diagnostic tool for PD. The prevalence of PD among Saudi adults was 13.1%. Most individuals with PD experienced their first panic attack before the age of 18. Only 38.3% individuals with PD sought medical attention, and approximately one-third of those who sought help did not receive a diagnosis. Multiple logistic regression analysis revealed that significant risk factors for PD included being female; having chronic health problems, a comorbid psychiatric disorder, a high body mass index; and experiencing suicidal ideation (*P* < 0.05). The highest risk was associated with chronic diseases (adjusted odds ratio = 3.1, 95% confidence interval: 2.1–4.6). This study demonstrates that PD is a prevalent and debilitating mental health condition among Saudi Arabian adults. Non-mental health physicians should be aware of PD, as many cases remain undiagnosed.

## Introduction

Anxiety disorders are a leading public health problem worldwide, affecting more than 301 million people, and ranking among the top 25 leading causes of years lived with disabilities (YLDs) [1].

Among anxiety disorders, panic disorder (PD) is considered one of the most debilitating and is characterized by recurrent unexpected panic attacks (PAs) and persistent worry about future attacks and their consequences. A PA is a sudden episode of intense fear that appears quickly and reaches its peak within minutes. During a PA, people experience various symptoms, including chest pain or discomfort, palpitations or accelerated heart rate, sweating, shortness of breath, choking, dizziness, trembling or shaking, abdominal discomfort or nausea, dissociative symptoms, and paresthesia. They may also feel that they are losing control or are dying. PD can have a significant impact on life. People with PD may avoid going to certain places or doing certain things for a fear of PAs. They may experience social isolation, decreased productivity, and impaired relationships [2].

PD, like other anxiety disorders, is associated with a range of comorbid conditions such as respiratory, cardiovascular, and gastrointestinal diseases [3]. Additionally, PD is comorbid with various psychiatric disorders, including depressive disorder, other anxiety disorders (such as generalized anxiety disorder, social phobia, agoraphobia, and posttraumatic stress disorder), substance use disorder, and a high suicidal ideation and attempt rates [4, 5].

Globally, the lifetime prevalence of PAs is 13.2%, with two-thirds of those experiencing recurrent PAs without meeting the diagnostic criteria for PD. The lifetime prevalence of PD is 1.7% [6]. A nationally representative study of the United States population aged 15–54 years found a 2.7% prevalence of PD over 12 months and a lifetime prevalence of 4.7% [7].

PD typically develops in early adulthood, with a mean age of onset of 30.3 years (95% confidence interval [CI]: 26.09–34.59) [8]. The prevalence of PAs and PD peaks in the 30–39 and 40–49 age groups, respectively, and declines thereafter. PD rarely develops for the first time after 60 years of age. Females were twice as likely to develop PD as males [9, 10].

Two recent studies of the 12-month prevalence, severity, lifetime prevalence, and age-of-onset distribution of mental disorders based on the Saudi National Mental Health Survey (SNMHS) data found that the 12-month prevalence of PD was 1.3% (standard error [SE]: 0.3%). These studies have classified the severity of PD into three categories: serious, moderate, and mild. Serious severity was defined as reporting severe impairment in at least two of the four functional domains or having attempted suicide in the past 12 months in conjunction with PD. Studies have found that 61.7% of patients with PD have serious severity (SE: 9.4%). The lifetime prevalence of PD in the SNMHS group was 1.6% (1.9% in females and 1.3% in males), with a peak prevalence of 2.3% in the 35–49 age group [11, 12].

Regarding predictive factors, a systematic review of cohort studies on the risk factors for PD onset in the general adult population found that age 20–54 years; female sex; low socioeconomic status; nicotine smoking during adolescence; alcohol use disorder; certain physical diseases such as cardiac diseases, migraine, and bronchial asthma; parental history of mental disorders; and the presence of other anxiety disorders were associated with PD onset. Ethnicity, educational level, marital status, and parental characteristics were not associated with the onset of PD [13]. High caffeine consumption increases anxiety and induces PAs in a large proportion of patients with PD [14].

PD is a common mental health condition that can have a significant impact on life. However, limited research has been conducted on the prevalence of PD and their associated factors in Saudi Arabia. This study aims to address this knowledge gap.

## Materials and methods

A cross-sectional online survey was conducted to estimate the prevalence of Panic Disorder (PD) among Saudi Arabian adults aged 18 years and older. The study was limited to Saudi nationals within this age group who provided informed consent to participate. Data were collected between July and October 2023 using Google Forms, which were distributed on social media platforms. The Patient Health Questionnaire-Panic Disorder (PHQ-PD) is a validated tool for screening and diagnosing PD [15, 16].

The PHQ-PD scale has been translated into Arabic and validated by Alhadi et al., with an acceptable internal consistency of α = 0.696 [17]. The PHQ-PD consists of two parts: screening and symptom checklist. The screening began with the question, “In the past four weeks, have you had a sudden attack of extreme fear or panic?” If the subject answered “Yes,” they were asked to complete three additional questions about the attack details. The symptom checklist consisted of 11 items that assessed the physical symptoms experienced during PAs. To diagnose a participant with PD, they must answer “yes” to all four questions in the screening and at least four positive symptoms from the 11 items in the symptom checklist.

The questionnaire used in our study was divided into three sections, the first of which included socio-demographic details such as age, gender, employment, nationality, and medical and psychological history. The second section included PD assessment using the PHQ-PD questionnaire. The third section included the PD pattern, frequency, age at onset, medical consultation history, nicotine smoking, physical exercise activities, body mass index, and substance abuse.

The questionnaire was validated in a pilot study of 15 participants to assess its clarity, validity, and reliability. A panel of two experts reviewed the questionnaire items to assess the content validity. The experts first reviewed the items independently, and then discussed any disagreements until a consensus was reached. All suggested changes were made to improve the validity of the questionnaire.

To address the potential sources of bias, the online questionnaire was limited to one response per participant. Participants were required to log in to their email addresses to begin the survey, and those with the same email address could not fill out the survey again. This helped ensure that the data were collected from a diverse range of individuals and that each participant only responded to the survey once.

To explore the prevalence of Panic Disorder (PD) among Saudi Arabian adults and assess associated predictive factors, we calculated a sample size of 385 respondents. This calculation was based on achieving a balance between precision and feasibility within the constraints of an online survey method, targeting a broader participation with a confidence level of 95% and a margin of error of 5%. Recognizing the limitations of non-probability sampling in fully capturing the diversity of the adult Saudi population, we aimed to exceed this minimum by recruiting at least 1000 respondents to enhance the robustness of our analysis.

The data were collected, reviewed, and entered into the Statistical Package for Social Sciences version 21 (SPSS, Inc., Chicago, IL, USA). All statistical methods used were two-tailed, with an alpha level of 0.05, considering significance if the p-value was less than or equal to 0.05. Regarding PD assessment, the PHQ criteria were applied, and the study adults were categorized into those fulfilling the criteria for having PD, and those who did not. Descriptive analysis was performed by prescribing the frequency distribution and percentage of study variables, including participants’ personal data, employment, medical data, psychological history, body mass index (BMI), and daily habits and behaviors. Additionally, the overall prevalence of PD was graphed, and the PD features and frequencies were tabulated. PD and its relationship with adults’ biodemographic characteristics and their daily activities and behaviors were cross tabulated by conducting the Pearson chi-square test for significance and exact probability test if there were small frequency distributions. A multiple logistic regression model was used to assess significant predictors of PD among adults with adjusted risk effects (adjusted odds ratio [AOR] and 95% CI).

## Results

This study surveyed 1,276 Saudi adults from different regions of Saudi Arabia. Participants’ ages ranged from 18 to 65 years, and most (77.4%) were aged 18–25 years. Females (68.4%) and students (61.1%) comprised the majority of the participants.

Overall, 167 (13.1%) participants met the criteria for PD. Of those with PD, 65.3% experienced one PA in the last four weeks, whereas 29.3% experienced two–four attacks. Additionally, 55.7% had their first PA before the age of 18 years, whereas 36.5% had their first attack between the ages of 18 and 25 years. PD significantly impacted the lives of those affected, with 45.5% experiencing high difficulty in working, studying, and carrying out responsibilities at home, and 13.8% experiencing extreme difficulty. Only 38.3% of the patients sought medical attention for their symptoms, of whom 36.8% visited a psychiatrist, 20.6% visited a family physician, and 16.2% visited an internal medicine specialist. Of those who visited a doctor for PAs, 6.6% were diagnosed with an organic medical disease that explained their symptoms, 61.7% were diagnosed with PD, and the remaining 31.7% were not diagnosed. Over 42.5% of patients with PD had medical comorbidities, and over 24.6% had comorbid psychiatric disorders (Table 1).

**Table 1:** Pattern, frequency, onset, effect, and comorbidities of panic disorder (PD) among Saudi adults (*n* = 167)

PD characteristics	No	%
How many times have you had a panic attack in the last four weeks?		
One time	109	65.3%
2–4 times	49	29.3%
5 or more times	9	5.4%
Age at the first onset of PD (years)		
< 18	93	55.7%
18–25	61	36.5%
26–35	4	2.4%
36–45	8	4.8%
45+	1	0.6%
How difficult has this problem made it for you to do your work, study, carry out your responsibilities at home, or even get along with people?		
Not difficult at all	9	5.4%
Somewhat difficult	59	35.3%
Very difficult	76	45.5%
Extremely difficult	23	13.8%
Have you visited any doctor because of these symptoms?		
Yes	64	38.3%
No	103	61.7%
What is the specialty of the doctor you visited for these symptoms? (*n* = 64)		
Psychiatrist	25	36.8%
Family medicine	14	20.6%
Internal medicine	11	16.2%
Cardiologist	7	10.3%
ER	7	10.3%
Others	4	5.9%
Have you been diagnosed by your doctor with a physical condition that accounts for your symptoms?		
Yes	11	6.6%
No	156	93.4%
Have you been diagnosed with PD?		
Yes	103	61.7%
No	64	38.3%
Have you had any suicidal ideation?		
Yes	34	20.4%
No	133	79.6%
How often do you engage in physical exercise?		
Never practice	90	53.9%
1–2 days / week	60	35.9%
3–4 days / week	13	7.8%
5–7 days / week	4	2.4%
Do you consume caffeine regularly?		
Yes	133	79.6%
No	34	20.4%
Do you currently use nicotine products such as cigarettes, e-cigarettes?		
Yes	21	12.6%
No	146	87.4%
Do you currently consume alcohol or use any illicit drugs?		
Yes	1	0.6%
No	166	99.4%
Medical Comorbidities		
None	96	57.5%
Diabetes mellitus	11	6.6%
Hypertension	8	4.5%
Irritable bowel syndrome	31	18.6%
Migraine	18	10.8%
Thyroid disorders	17	10.2%
Cardiac diseases	1	0.6%
Respiratory diseases	9	5.4%
Others	3	1.8%
Psychiatric Comorbidities		
None	126	75.4%
Depression	22	13.1%
Social phobia	17	10.2%
Generalized anxiety disorder	17	10.2%
Obsessive–compulsive disorder	1	0.6%
Agoraphobia	1	0.6%

Table 2. shows PD and its relationship with adults biodemographic characteristics, medical comorbidities, and psychiatric comorbidities. We found that 14.3% of all female participants had PD compared with 10.4% of males with recorded statistical significance (*P* = 0.048). Furthermore, PD was detected among 44.7% of those with thyroid disorders, 37.5% of the adults with migraine attacks, 32% of those with irritable bowel syndrome, 30.8% of those with hypertension, compared with 9.2% of those with no chronic health problem (*P* = 0.001). Similarly, 44.7% of the adults with social phobia experienced PD compared with 35.5% of those with depression and only 10.9% of adults with no psychological disorder (*P* = 0.001). PD was also detected among 28.7% of obese adults compared with 13.2% of those with overweight and only 9.8% of adults with normal weight (*P* = 0.001).

**Table 2:** Panic disorder and its relationship with adults’ biodemographic characteristics and medical and psychiatric comorbidities

Bio-demographics	Total		Panic Disorder				p-value
			Yes		No		
	Number	%	Number	%	Number	%	
**Age in years**							0.370
18–25	987	77.4%	121	12.3%	866	87.7%	
26–35	174	13.6%	26	14.9%	148	85.1%	
36–45	65	5.1%	12	18.5%	53	81.5%	
45+	50	3.9%	8	16.0%	42	84.0%	
**Gender**							0.048*
Male	403	31.6%	42	10.4%	361	89.6%	
Female	873	68.4%	125	14.3%	748	85.7%	
**Employment Status**							0.284
Unemployed	288	22.6%	35	12.2%	253	87.8%	
Student	779	61.1%	102	13.1%	677	86.9%	
Education sector	69	5.4%	10	14.5%	59	85.5%	
Health care sector	55	4.3%	12	21.8%	43	78.2%	
Military sector	85	6.7%	8	9.4%	77	90.6%	
**Medical Co-morbidities**							0.001*^
None	1046	82.0%	96	9.2%	950	90.8%	
Diabetes mellitus	43	3.4%	11	25.6%	32	74.4%	
Hypertension	26	2.0%	8	30.8%	18	69.2%	
Irritable bowel syndrome	97	7.6%	31	32.0%	66	68.0%	
Migraine	48	3.8%	18	37.5%	30	62.5%	
Thyroid disorders	38	3.0%	17	44.7%	21	55.3%	
Cardiac diseases	5	0.4%	1	20.0%	4	80.0%	
Respiratory diseases	29	2.3%	9	31.0%	20	69.0%	
Others	18	1.4%	3	16.7%	15	83.3%	
**Psychiatric Co-morbidities**							0.001*^
Never	1152	90.3%	126	10.9%	1026	89.1%	
Depression	62	4.9%	22	35.5%	40	64.5%	
Social phobia	38	3.0%	17	44.7%	21	55.3%	
Generalized anxiety disorder	52	4.1%	17	32.7%	35	67.3%	
Obsessive-compulsive disorder	15	1.2%	1	6.7%	14	93.3%	
Agoraphobia	5	0.4%	1	20.0%	4	80.0%	
**Body mass index**							0.001*
Normal (body–mass index [BMI]: 18.5–24.9)	762	59.7%	75	9.8%	687	90.2%	
Overweight (BMI: 25–29.9)	357	28.0%	47	13.2%	310	86.8%	
Obese (BMI: 30 or more)	157	12.3%	45	28.7%	112	71.3%	

P: Pearson X^2^ test ^: Exact probability test

* *P* < 0.05 (significant)

Table 3. shows PD and its relationship with adults’ daily habits and behaviors. We found that 16.5% of adults who never practice physical exercise experienced PD versus 6.2% of others who practice for 5–6 days per week (*P* = 0.008). Furthermore, 15.3% of non-smokers experienced PD compared with 6.6% of smoking adults (*P* = 0.001). PD was detected among 38.5% of adults who had suicidal ideation during the last four weeks compared with 11.3% of those who did not (*P* = 0.001).

**Table 3:** Panic disorder and its relationship with adults’ daily habits and behaviors

Habits and Behaviors	Total		Panic Disorder				p-value
			Yes		No		
	Number	%	Number	%	Number	%	
**Physical Exercise**							0.008*
Never practice	546	42.8%	90	16.5%	456	83.5%	
1–2 days / week	514	40.3%	60	11.7%	454	88.3%	
3–4 days / week	151	11.8%	13	8.6%	138	91.4%	
5–7 days / week	65	5.1%	4	6.2%	61	93.8%	
**Drinking coffee**							0.270
Yes	973	76.3%	133	13.7%	840	86.3%	
No	303	23.7%	34	11.2%	269	88.8%	
**Nicotine Smoking**							0.001*
Yes	320	25.1%	21	6.6%	299	93.4%	
No	956	74.9%	146	15.3%	810	84.7%	
**Substance Abuse**							0.624^
Yes	12	0.9%	1	8.3%	11	91.7%	
No	1264	99.1%	166	13.1%	1098	86.9%	
**Suicidal Ideation**							0.001*
Yes	95	7.4%	34	35.8%	61	64.2%	
No	1181	92.6%	133	11.3%	1048	88.7%	

P: Pearson X^2^ test ^: Exact probability test

* *P* < 0.05 (significant)

Table 4 shows the results of multiple logistic regression analysis to identify the predictors of PD among Saudi adults. After controlling for all other factors, female sex, chronic health problems, psychiatric disorders, high BMI, and suicidal ideation were significant risk factors for PD. Smoking was the only significant protective factor identified. The highest risk was associated with having chronic diseases (AOR = 3.1, 95% CI: 2.1–4.6), followed by suicidal ideation (AOR = 2.8, 95% CI: 1.7–4.7) and having psychiatric disorders (AOR = 2.3, 95% CI: 1.4–3.6).

**Table 4:** Multiple logistic regression analysis for predictors of panic disorder among Saudi adults

Predictors	p-value	AOR	95% CI	
			Lower	Upper
Age	0.354	0.9	0.7	1.1
Gender	0.032*	1.6	1.0	2.3
Employment status	0.879	1.0	0.8	1.2
Medical Co-morbidities	0.001*	3.1	2.1	4.6
Psychiatric Co-morbidities	0.001*	2.3	1.4	3.6
Body–mass index	0.004*	1.4	1.1	1.9
Physical Exercise	0.491	1.1	0.8	1.7
Have coffee	0.162	1.4	0.9	2.1
Smoking	0.024*	0.51	0.30	0.99
Substance Abuse	0.843	0.8	0.1	6.6
Suicide ideation	0.001*	2.8	1.7	4.7

AOR: Adjusted odds ratio CI: confidence interval

* *P* < 0.05 (significant)

## Discussion

This study investigated the prevalence of PD and associated factors among the adults of Saudi Arabia. This study’s findings have important implications for helping healthcare providers better understand the burden of PD and develop effective prevention and treatment strategies. The findings revealed a significant prevalence of PD among Saudi adults, with an estimated 13.1% of the participants meeting the diagnostic criteria. The prevalence in our study was higher than that reported by Altwaijri et al. (1.9%) [11] and compared to the annual incidence of PD reported by the The European Study of the Epidemiology of Mental Disorders (i.e., 0.8% in Europe and 0.6% in Spain, with lifetime prevalence of 2.1% and 1.7%, respectively) [18]. A systematic review conducted by Habadi et al. on the prevalence of PD in primary healthcare, encompassing 6,651 patients, ranged from 10.3 to 1.2%, with a weighted prevalence of 5% [19]. A nationally representative study in the United States population found that the lifetime prevalence of PD was 4.7% [7]. Kessler RC reported that the lifetime prevalence of PAs was 22.7%, that of PD without agoraphobia was 3.7%, and that of PD with agoraphobia was 1.1% [20].

The higher prevalence estimates in our study compared with those in other studies may be attributed to methodological differences, as we used self-report questionnaires instead of structured interviews. Self-reported psychological tests can provide higher percentages than structured interviews for diagnostic purposes. Structured interviews are more specific, identifying individuals with plausible symptoms of a disorder correctly by discouraging non-psychiatric reports and encouraging the identification of psychiatrically relevant symptoms [21].

In our sample, 59.3% of the individuals with PD reported that their PD affected them highly or extremely. This is consistent with the findings of Kessler et al., who reported that moderate-to-severe scores were observed in 86.3% of individuals with panic disorder with agoraphobia, 46.1% of individuals with panic disorder without agoraphobia, and 6.7% of individuals with isolated panic attacks [20].

According to Tibi et al., the age of onset of PD can be divided into early age of onset (onset at 27 years old or less) and late age of onset (onset after 27 years old). In their study, the early onset group comprised 62% of participants [22]. The mean age at onset was 17 years (SD = 6.9 years) in the early onset population and 39 years (SD = 9.4 years) in the late-onset population [22]. Consistent with this finding, our study found that PD has an early onset, with 55.7% of participants experiencing the onset before the age of 18 years. This early onset pattern may be related to the young age distribution of our sample, with 77.4% of the participants falling within the 18–25 age group. This finding suggests that PD is more prevalent in younger individuals. Furthermore, the current study revealed that females were more likely to develop PD than males which is consistent with the existing literature’s findings [9, 10].

People with PD in the United States are more likely to seek treatment within one year of onset than those with generalized anxiety disorder, specific phobia, or social anxiety disorder. The median delays for first treatment contact for these other anxiety disorders were 1 year, 13 years, and 16 years [23]. Longer duration of untreated PD (> 1 year) may be a predictor of a comorbid major depressive disorder (MDD) [24].

Patients with PD often seek treatment from a variety of specialists, including primary care physicians, emergency room doctors, and cardiologists, depending on the nature of their symptoms and availability of care [25]. Approximately 8% of patients seen by cardiologists presented PD symptoms. Moreover, 10% of the patients who undergo coronary artery bypass surgery may also have PD. Among the patients with chest pain and normal angiography findings, up to 30% also had PD. Many patients with PD continue to believe that they have heart disease even after diagnosis, and may not seek help from psychiatrists despite having disabling symptoms and continuing to use emergency room services [26].

The incidence of PD in patients with chronic obstructive pulmonary disease is significantly elevated and is up to ten times higher than that in the general population [27]. Similarly, 15% of patients with dizziness in ENT clinics had PD [28]. Up to 40% of the patients with gastrointestinal presentations in primary healthcare settings develop PD [29]. Individuals with migraine had an approximately four-fold increased risk of developing PD, with the highest rates observed in those with migraine and aura. However, such patients may remain undiagnosed [30].

We found that over 42.5% of patients with PD had medical comorbidities, mainly irritable bowel syndrome (18.6%), migraine (10.8%), and thyroid disorders (10.2%). Similarly, PD was detected in 44.7% of those with thyroid disorders, 37.5% of adults with migraine attacks, 32% of those with irritable bowel syndrome, and 30.8% of those with hypertension.

In our study, only 38.3% of the patients who met the criteria for PD sought medical attention, primarily from non-mental health physicians. Approximately one-third of those who met the criteria for PD and consulted a physician did not receive a diagnosis of PD or general medical condition that could explain their symptoms. This suggests that most patients with PD in Saudi Arabia do not seek medical attention. Therefore, primary care physicians, internists, cardiologists, emergency physicians, and other medical specialists should be more familiar with PD and identify it early to reduce the burden of untreated illnesses and unnecessary repeat investigations.

According to our study, over 24.6% of the patients with PD had comorbid psychiatric disorders. The most common psychiatric comorbidities were depressive disorder (13.1%), social phobia (10.2%), and generalized anxiety disorder (10.2%). Similarly, 44.7% of adults with social phobia had PD, compared with 35.5% of those with depressive disorder. Similar results were found in different studies showing that MDD and PD are frequently comorbid, with up to half of the individuals having a lifetime history of MDD and a history of PD. Individuals with MDD are 19 times more likely to develop PD than those without MDD. Additionally, untreated PD may increase the risk of developing MDD [31, 32].

In our study, we found a high rate (20.4%) of suicidal ideation among patients with PD. Furthermore, suicidal ideation increased the risk of PD (AOR = 2.8, 95% CI = 1.7–4.7). Sareen reported that individuals with PD have a 7.85-fold increased risk of developing suicidal ideation and suicide attempts compared with those without PD [33]. This highlights the importance of screening all individuals with PD for suicidal ideation and providing appropriate treatment and support.

In our study, PD was detected among 28.7% of obese adults compared to 9.8% of adults with normal BMI. Simon et al. found that obese individuals have a 27% higher lifetime risk of developing PD than non-obese individuals [34]. The increased risk of developing PD in obese individuals may be attributed to lifestyle factors and medical comorbidities in obese patients. Clinicians should be aware that obese adults have an increased risk of PD and should screen them for PD in primary care and obesity clinics as PD can affect the outcome of medical interventions. Additionally, weight loss interventions may help reduce the risk of PD in obese adults. Further research is needed to understand the mechanisms linking obesity and PD to develop more effective preventive and treatment strategies.

In our study, we found that increasing the frequency of exercise was associated with low PD occurrence; 16.5% of adults who never engaged in physical exercise had PD versus 6.2% of those who exercised for 5–6 days per week. Other studies have reported similar associations between exercise and PD. A randomized controlled trial found that exercise is less effective than cognitive behavioral therapy in reducing panic and agoraphobic symptoms. However, exercise remains beneficial because it significantly reduces PD symptoms [35]. A different randomized controlled trial found that people with PD had clinically significant improvements in panic symptoms after a 14-day exercise intervention that included six 20-minute sessions of high-intensity exercise [36]. People who performed a single session of high-intensity exercise after inhaling 35% carbon dioxide to induce a PA, had significantly reduced panic symptoms [37]. Another study found that exercise effectively reduced anxiety symptoms in patients with PD, with a moderate effect size [38].

In our study, smoking was found to be a protective factor against PD, even though smoking is a known risk factor of PD and can worsen PAs [39]. This can be explained by a preclinical study that concluded that nicotine inhibits beta-2* nicotinic acetylcholine receptors (nAChRs). The stimulation of these receptors can lead to increased anxiety-like behaviors. Low-dose nicotine exposure can inhibit these receptors and prevent the increase in anxiety-like behavior that is caused by high doses of nicotine or other cholinergic agents [40]. Although our result is statistically significant, the sample size of our study was relatively small (only 21 persons diagnosed with PD were smokers), which limits the power of our findings. Additionally, smoking may be used as a coping mechanism to prevent PAs. Smoking can distract people from anxiety-provoking thoughts and feelings, which may reduce their risk of developing PD. However, it is important to note that smoking is an addictive substance with many other negative health consequences, and further research is needed to confirm this result.

This study has several limitations. First, most participants were young adults, which may restrict the generalizability of the findings to older age groups. It is plausible that the observed associations between PD and the identified risk factors differ in older adults. Second, the use of self-report questionnaires may have introduced some degree of bias into the results. Participants may have been more inclined to report symptoms if they were aware of the study’s focus on PD. Third, the cross-sectional design of the study constrains its ability to establish causal inferences. Fourth, while this study provides valuable insights into the prevalence of Panic Disorder (PD) among Saudi Arabian adults, it did not assess the geographical distribution of participants, limiting our ability to analyze potential regional differences in PD prevalence and associated risk factors within the Kingdom. Despite these limitations, the study also has several strengths. First, it employed a validated diagnostic tool to assess the prevalence of PD among a large sample of Saudi adults. Second, it utilized a comprehensive set of statistical methods to identify potential risk factors associated with PD. Third, it provides valuable insights into the prevalence of PD and its associated factors in Saudi Arabia, a region where research on this topic is limited.

Overall, this study carries significant theoretical implications. It highlights the need for increased awareness and education about PD among the general public and healthcare professionals, as well as the importance of preventive interventions and targeted screening programs. Additionally, the study underscores the need for improved access to mental health services in Saudi Arabia, with a particular emphasis on educating non-mental health physicians about the symptoms of PD and enabling them to refer patients for appropriate care. Further research is warranted to investigate the long-term progression of PD in Saudi Arabia and identify predictors of treatment outcomes. Additionally, studies should explore the reasons why many cases of PD go undetected and undiagnosed by healthcare providers, thereby facilitating the development of strategies to address these barriers to diagnosis and treatment.

### Conclusions and Recommendations

In conclusion, this study found that PD is common among adults in Saudi Arabia, particularly among young women. People who are obese, have low physical activity levels, suicidal ideation, or comorbid medical or psychiatric conditions are at an increased risk of developing PD. PD typically starts early in life and can have a significant impact on daily activities, placing a burden on patients and their families. Non-mental health physicians should be aware of PD as many patients are not diagnosed. Therefore, any patient who expresses recurrent and pervasive worry, or presents with unexplained physical symptoms, should be evaluated for PD. Future research should explore the reasons why many cases of PD go undetected and undiagnosed by healthcare providers.

## Data Availability

No datasets were generated or analysed during the current study.

## Abbreviations

PA: Panic Attack
PD: Panic Disorder
SE: Standard Error
SNMHS: Saudi National Mental Health Survey
AOR: Adjusted Odds Ratio
BMI: Body Mass Index
PHQ-PD: The Patient Health Questionnaire-Panic Disorder
